# Temporal and geographical external validation study and extension of the Mayo Clinic prediction model to predict eGFR in the younger population of Swiss ADPKD patients

**DOI:** 10.1186/s12882-017-0654-y

**Published:** 2017-07-17

**Authors:** Laura Girardat-Rotar, Julia Braun, Milo A. Puhan, Alison G. Abraham, Andreas L. Serra

**Affiliations:** 1 Epidemiology, Biostatistics and Prevention Institute, University of Zurich, Hirschengraben 84, 8001 Zurich, Switzerland; 2 Johns Hopkins University School of Medicine, Baltimore, MD USA; 3 Medizinisches Kompetenzzentrum für ADPKD, Suisse ADPKD, Department of Internal Medicine and Nephrology, Hirslanden, Witellikerstrasse 40, CH-8032 Zurich, Switzerland

**Keywords:** ADPKD, Disease progression, Epidemiology, Kidney volume, Prediction model, Validation study

## Abstract

**Background:**

Prediction models in autosomal dominant polycystic kidney disease (ADPKD) are useful in clinical settings to identify patients with greater risk of a rapid disease progression in whom a treatment may have more benefits than harms. Mayo Clinic investigators developed a risk prediction tool for ADPKD patients using a single kidney value. Our aim was to perform an independent geographical and temporal external validation as well as evaluate the potential for improving the predictive performance by including additional information on total kidney volume.

**Methods:**

We used data from the on-going Swiss ADPKD study from 2006 to 2016. The main analysis included a sample size of 214 patients with Typical ADPKD (Class 1). We evaluated the Mayo Clinic model performance calibration and discrimination in our external sample and assessed whether predictive performance could be improved through the addition of subsequent kidney volume measurements beyond the baseline assessment.

**Results:**

The calibration of both versions of the Mayo Clinic prediction model using continuous Height adjusted total kidney volume (HtTKV) and using risk subclasses was good, with R^2^ of 78% and 70%, respectively. Accuracy was also good with 91.5% and 88.7% of the predicted within 30% of the observed, respectively. Additional information regarding kidney volume did not substantially improve the model performance.

**Conclusion:**

The Mayo Clinic prediction models are generalizable to other clinical settings and provide an accurate tool based on available predictors to identify patients at high risk for rapid disease progression.

## Background

Prediction models in autosomal dominant polycystic kidney disease (ADPKD) are used in clinical settings for several purposes. They can inform patients about their prognosis. They can identify patients at greatest risk of rapid disease progression who might benefit most from new therapies. They can also identify patients with slower disease progression who might benefit from a care strategy that delays treatment until a later stage [1, 2]. Finally, prediction models are useful for identifying patients with a particular disease risk profile who would be suitable for clinical trials [2, 3]. Relevant outcomes for ADPKD prediction models include total kidney volume (TKV) and estimated glomerular filtration rate (eGFR) [4], the primary clinical indicators of disease progression. Established predictors of these outcomes include age, sex, earlier measures of TKV and eGFR and Polycystic Kidney Disease genotype [5, 6].

The vasopressin V2 receptor antagonist, tolvaptan, has been recently approved for the treatment of ADPKD but, due to notable side effects and expense, represents a treatment where good risk prediction is important for targeting use. Tolvaptan is the first approved drug shown to directly affect disease progression [7]; all other therapies target co-morbidities that may contribute to progression but do not affect the underlying disease [8]. The indication for tolvaptan is currently limited to patients with evidence of rapid progression in Switzerland and European Union according to the European Medicines Agency [9] where the expected benefit outweighs the risk of side effects and associated high treatment costs [10]. The challenge for clinicians is to identify patients at highest risk of rapid progression without extensive diagnostic screening across the full patient population. Currently, TKV and the rate of TKV change are considered the most accurate predictors of progression [11]. However, for routine clinical and research purposes, direct measurement of kidney volume is less feasible due to time and technical demands as well as expense.

Recently, Mayo Clinic investigators developed a risk classification system for ADPKD patients using a single TKV value and an accompanying prediction model [12, 13]. In 2016, the European Renal Association – European Dialysis and Transplant Association Working Group published a recommendation that the Mayo Clinic prediction model be used to discriminate patients at high risk for rapid disease progression [14]. However, the prognostic performance of the prediction model has yet to be evaluated in an external sample outside the US, which is critical for establishing accuracy and generalizability of risk discrimination across different patient populations [12].

The aim of our study was to externally validate the Mayo Clinic Model using data from the prospective longitudinal Swiss ADPKD study, with a patient population both geographically and temporally removed from the original patient population in which the model was developed. We also sought to evaluate whether improved prediction performance could be achieved by including additional measurements of the most relevant predictor: height adjusted total kidney volume (HtTKV).

## Methods

### Swiss ADPKD validation data

Participants were eligible for the Swiss ADPKD study if they had an ADPKD diagnosis, were over 18 years of age and had an eGFR over 30 ml per min per 1.73m^2^ at enrolment [15]. For the present analysis, participants from the Swiss ADPKD study were included if they were under active follow-up between 2006 to 2016, had at least one follow-up visit and had not been treated with tolvaptan. Approximately 3% (*N* = 6) of patients had Atypical ADPKD (Class 2) and were excluded from the present analysis. Visits were done at the university hospital in Zurich and at the Hirslanden hospital Zurich. At every scheduled clinical visit, data were collected on medical history, kidney imaging metrics and laboratory values from blood and urine samples. Clinical measurements and assays were done according to a protocol with standardized operating procedures [16, 17]. Following an initial visit, a second visit occurred within 6–12 months and then visits were scheduled annually; when a study participant missed a scheduled visit, a study visit occurred at the next available opportunity to collect Magnetic Resonance Imaging (MRI) and other study data. The local ethics committee in Zurich approved the study (EK-number 1178) and all patients provided written informed consent.

### Mayo Clinic risk classes and eGFR prediction model

**Fig. 1:**
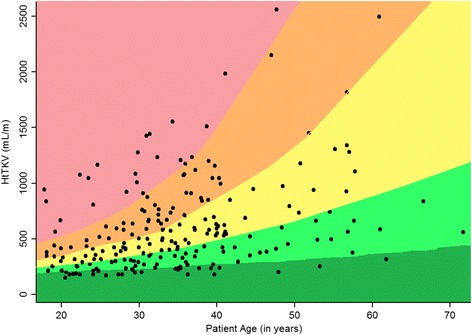
Subclassification of ADPKD patients based on HtTKV limits on their age at baseline. Limits are defined from the Mayo Clinic based on estimated kidney growth rates of <1.5% (*dark green*), 1.5–3.0% (*mint*), 3–4.5% (*yellow*), 4.5–6% (*orange*) and >6% (*red*)

The Mayo Clinic prediction model has been described [12]. Briefly, five risk subclasses with theoretical yearly percentage increases in kidney volume of <1.5% (Class 1A), 1.5–3% (Class 1B), 3–4.5% (Class 1C), 4.5–6% (Class 1D) and >6% (Class1E) were defined based on age and imaging data (Fig. 1) [12]. Then a linear mixed-effect model was used to predict eGFR after *t* years of follow-up using baseline (*t* = 0) predictors: log_2_HtTKV or risk subclass group (1A-1E) [12], sex, age, eGFR from the Chronic Kidney Disease Epidemiology Collaboration (CKD-EPI) equation [18]. Years of follow-up was included as a linear term with a subject specific random effect. Interaction terms of years of follow-up with all predictors were also included in the model.

### Outcome

#### Estimated glomerular filtration rate (eGFR)

In accordance with the Mayo Clinic model, our primary outcome was eGFR at *t* years follow-up. Serum creatinine was measured at each visit by the central laboratory institute of clinical chemistry of the university hospital and the central laboratory in Zurich using the modified Jaffé method traceable to an isotope-dilution mass spectroscopy reference [17]. eGFR at baseline (*t* = 0) was estimated using the CKD-EPI equation [18].

### Predictors

#### Total kidney volume (TKV)

At every study visit, a measurement of kidney volume was taken by using a standardized procedure protocol for MRI’s [16]. MRI acquisitions contain a breath-hold T1-weighted fast spoiled gradient echo sequence without fat suppression sequence (4 mm slice thicknesses) and trans-axial T2 weighted fast spin echo sequences. TKV was estimated by hand contouring [16]. Height adjusted total kidney volume (HtTKV) was obtained by dividing TKV by patient height (ml/m).

#### Statistical methods

Baseline characteristics are given as proportions and medians (interquartile range). Patients were stratified into the five subclasses (1A-1E) based on the Mayo Clinic estimated kidney growth rates limits of 1.5%, 3.0%, 4.5% and 6% (Fig. 1).

We applied the Mayo Clinic model to all participants of the Swiss ADPKD study to predict eGFR at *t* years follow-up, using log_2_HtTKV as a continuous predictor and keeping regression coefficients fixed at the values determined from the Mayo Clinic development sample. We also applied a second Mayo Clinic model, that replaced the baseline (*t* = 0) log_2_HtTKV with baseline risk subclass (1A-1E).

To try to improve upon the Mayo equation predictive performance, we tested two modifications to the original Mayo model. First, we included in the model a second HtTKV follow-up measurement (mostly within 6–12 month of the baseline measurement) to provide additional information on individual change in TKV (Model 1). The regression coefficient for the HtTKV term was refit to the Swiss ADPKD study sample, but all other regression coefficients were kept fixed at their original value, including the intercept. Second, we included information on all subsequent and available HtTKV measurements, again refitting the regression coefficient for the HtTKV term while keeping all other regression coefficients fixed at their original Mayo Clinic values (Model 2). Updated models with and without interaction terms of HtTKV*years were evaluated.

#### Evaluation of model performance

The model fit to the validation data set was assessed using R-squared statistics and Akaike’s information criterion (AIC). Discrimination was visually assessed using scatter plots comparing observed and predicted eGFR values on the natural scale with an estimated regression line, line of equality and confidence interval. The agreement was assessed using the Bland-Altman analysis [19]. The bias, defined as average (observed eGFR – predicted eGFR) was estimated along with the 95% limits of agreement, defined as the bias ±1.96 standard deviation of the difference between observed and predicted eGFR. Lastly, the % of predicted eGFR within 30% of the observed eGFR was calculated. We followed Steyerberg’s approach to validate and update clinical prediction models [10].

To compare the performance of the prediction models we estimated the continuous ranked probability score (CRPS) of the 3 competing models: original model, updated model 1 and updated model 2. The CRPS is a proper scoring rule to assess univariate predictive distributions with smaller values indicating better predictive performance [20]. The metric takes into account the entire predictive distribution of the outcome [21] and assesses both calibration and precision of predictive distribution. For evaluation of models with added TKV information, five-fold cross-validation was used given that no external validation was available [10].

The predictor HtTKV was missing in 3% of the participant-visits. We used multiple imputation (MI) to impute the missing values; specifically, a Markov Chain Monte Carlo method was implemented and multivariate normality was assumed [22]. We generated 30 imputed data sets for each model with HtTKV [23].

Stata 13.1 was used for all data analysis and graphics.

## Results

### Characteristics of the Swiss ADPKD study sample

Between April 2006 and March 2016, 214 patients with an ADPKD diagnosis were enrolled in the Swiss ADPKD study, contributing a total of 1985 person-visits. At baseline, the median age was 34 years (interquartile range [IQR]: 27–40), the median eGFR was 82 ml/min per 1.73 m2 (IQR: 70–95) and the median HtTKV was 497 ml/m (IQR: 317–762). Swiss ADPKD study follow-up times ranged from a minimum of 0.42 years for new enrolees to a maximum time of 10.28 years. We assessed change from one class to another in 206 patients from the 214 swiss ADPKD class1 patients. In total, there were 52 patients (25%) from the 206 Swiss ADPKD Study participants who progressed to a more severe risk class over the median 5 year follow-up and 7 (3%) who changed to a milder disease risk class. More than the half of the patients in class 1A (56%) remained in their class: 40% (10 patients) progressed from 1A to 1B (1 patient from 1A to 1C), 18% (9 patients) progressed from 1B to 1C (1 patients from 1B to 1D), 28% (16 patients) progressed from 1C to 1D (4 patients from 1C to 1B) and 28% (15 patients) progressed from 1D to 1E (3 patients from 1D to 1C).

### Comparison of the Swiss ADPKD study sample to the Mayo development sample

Compared to the Mayo clinic development sample of 376 patients, the average eGFR was higher by 11 ml/min per 1.73 m^2^, median age was 10 years younger, and median HtTKV was 155 ml/m lower in the Swiss ADPKD study patients. Swiss ADPKD patients had a median follow-up time of 5 years (IQR: 2 to 9 years) compared to 6 years (IQR 4–10) in the Mayo Clinic patients. Comparing progression rates, more patients progressed in the Swiss ADPKD Study at 25% across all initial risk classes compared to 11% to 16% in the Mayo clinic development sample, though the median follow-up was 5 years compared to 4 years in the Mayo Clinic.

### External validation of the Mayo Clinic model

In the Swiss ADPKD patient group, the Mayo Clinic model with the predictor log_2_HtTKV performed well with explained variance (R^2^) of 78% (Table 2), compared to the R^2^ of 69% in the development data set. Replacing baseline TKV with risk subclasses in the model resulted in a poorer model fit with a R^2^ of 70%, which is slightly lower to the R^2^ of 72% noted in the development set.

**Fig. 2:**
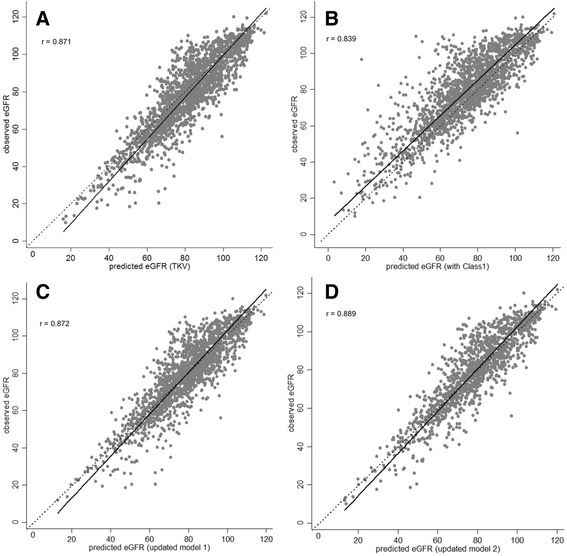
**a** Scatterplot of the observed eGFR versus the predicted eGFR derived from the model obtained from the development set with TKV as predictor with regression line and the line of equality. **b** Scatterplot observed eGFR vs. predicted eGFR derived from the model obtained from the development set with the five subclasses as predictor. **c** Scatterplot of the observed eGFR versus the predicted eGFR derived from the updated model 1 with two TKV measurements and **d** updated model 2 with time-varying TKV

**Fig. 3:**
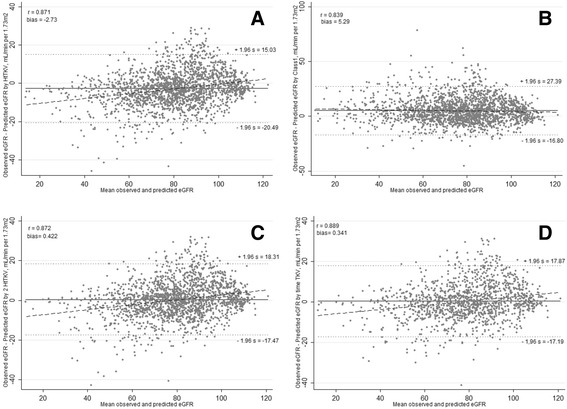
**a** Bland-Altman analysis of the observed eGFR versus the predicted eGFR derived from the model obtained from the development set with TKV as predictor. **b** Bland-Altman analysis observed eGFR vs. predicted eGFR derived from the model obtained from the development set with the five subclasses as predictor. **c** Bland-Altman analysis of the observed eGFR versus the predicted eGFR derived from the updated model 1 with two TKV measurements and **d** updated model 2 with time-varying TKV

The scatter plot of observed eGFR versus predicted eGFR indicated good discrimination with 91.5% of the predicted within 30% of the observed when log_2_HtTKV was included as a continuous predictor (Fig. 2a, Table 1) and 88.7% of the predicted within 30% of the observed when risk subclasses were included (Fig. 2b, Table 2). The Bland-Altman analysis shown in Fig. 3 indicated a lower bias for the log_2_HtTKV model and little distortion of the variability of the distribution, as seen from the approximate zero slope of the regression line.

**Table 1:** Baseline characteristics of the development set from the Mayo Clinic and the validation set from the Swiss ADPKD study

Class	Total number # (%)	Men/Women(n/n)	Median age (yr)	Median eGFR (ml/min per 1.73 m2)	Median HtTKV (mL/m)	Median follow-up (yr)
Mayo Clinic						
1A	40 (10.6)	7/33	50 (44–58)	84 (64–97)	249 (214–280)	5 (3–10)
1B	88 (23.4)	23/65	46 (36–53)	78 (62–97)	433 (322–565)	6 (3–9)
1C	122 (32.4)	46/76	44 (36–50)	71 (47–98)	701 (514–1037)	6 (4–10)
1D	77 (20.4)	40/37	41 (34–49)	60 (36–96)	1195 (843–1544)	6 (4–11)
1E	49 (13.0)	28/21	36 (29–43)	46 (26–94)	1874 (1118–2609)	5 (3–8)
Subtotal	376 (100)	144/232	44 (35–51)	71 (44–97)	651 (431–1195)	6 (4–10)
Swiss ADPKD						
1A	27 (12.6)	9/18	29.43 (24–37)	86.67 (78–102)	199.52 (178–232)	5.19 (1.99–8.35)
1B	52 (24.3)	20/32	36.15 (29–46)	83.84 (72–100)	343.75 (272–421)	5.28 (3.13–7.92)
1C	60 (28.0)	35/25	35.56 (28–41)	83.14 (71–92)	514.31 (407–630)	4.24 (2.02–8.26)
1D	52 (24.3)	38/14	32.48 (28–38)	79.35 (72–94)	705.70 (579–910)	6.25 (2.85–8.95)
1E	23 (10.7)	18/5	29.82 (23–35)	70.30 (56–86)	1166.50 (920–1425)	6.93 (3.27–8.53)
Subtotal	214 (100)	120/94	34.22 (27–40)	82.20 (70–95)	496.58 (317–762)	5.13 (2.21–8.48)

**Table 2:** Predictive performance of the validation, the updated models and the sensitivity analysis (in bold)

	R^2^	Bias (mL/min per 1.73 m^2^)^a^	95% Limits of Agreement (mL/min per 1.73 m2)^b^	Correlation	P30 (%)^c^	AIC	CRPS
Validation model: risk class (1985 observations)	0.7039	5.29	−16.8,27.3	0.839	88.7	–	–
Validation model: TKV (1985 observations)	0.7853	−2.73	−20.4,15.0	0.871	91.5	14,386.98	73.36
Updated model 1: 2 TKV’s (1867 observations)	0.7704	0.42	−17.4,18.3	0.872	96.6	13,557.91	58.16
*Updated model 1 with interaction term*	*0.7720*	*0.82*	*−16.9,18.6*	*0.879*	*96.6*	*1322.3*	*81.85*
Updated model 2: TKV time-varying (1344 observations)	0.7989	0.34	−17.1,17.8	0.889	96.1	9706.15	57.24
*Updated model 2 with interaction term*	*0.8015*	*0.57*	*−16.8,17.9*	*0.895*	*96.1*	*9715.05*	*83.39*

^a^Bias = average (observed eGFR – predicted eGFR)

^b^95% Limit of agreement = bias ±1.96*standard deviation of (observed eGFR – predicted eGFR)

^c^P30 = percentage of predicted eGFR within 30% of observed GFR (% within 30%)

*AIC* Akaike information criterion

*CRPS* continuous ranked probability score

### Improving the Mayo prediction model

To evaluate whether the Mayo prediction model performance could be improved if additional information on TKV was available, we modified the formula to include subsequently TKV measurements. Updated model 1 (number of observation = 1867), which included a follow-up TKV measurement, showed good overall performance with a R^2^ of 77%, an AIC of 13,557.91 and CRPS of 58.16 (Table 2). In updated model 2 (number of observation = 1344), which included all available follow-up TKV measurements, resulted in a slightly better CRPS of 57.24 and substantially improved AIC of 9706.15 compared to Model 1. The R^2^ was reasonably similar between the updated models and similar to the original Mayo Clinic model. Good agreement between observed and predicted was maintained as shown by the high correlation (Fig. 2c, d). Both updating models reduced the bias and provided a good fit to the data (Fig. 3c, d). An interaction term of TKV*years in the updated models did change performance (Table 2).

## Discussion

Accurate risk prediction is important for guiding clinical care, particularly when there are substantial costs to treatment. The goal of the Mayo Clinic model was to provide risk prediction for the ADPKD patient population; however prognostic performance has never been established in a broader patient sample and external validity of a prediction model is critical to assure accurate prediction across patient populations and therefore establish the model’s utility as a clinical tool.

Our results indicated that the Mayo Clinic model performs well in our Swiss ADPKD patient sample. Both models showed adequate discrimination and good calibration. The overall prediction performance in our sample as assessed with R^2^ was higher when the continuous predictor HtTKV was the used than when risk subclasses were used. These results suggest the models are generalizable and would perform well in routine clinical settings. Given the higher eGFRs in the Swiss ADPKD Study, these results were particularly notable, as poorer performance might be expected with upward shifts in the distribution of eGFR compared to the development set. However it should be noted that in the original Mayo Clinic prediction model and in our validation, an estimated eGFR from the CKD-Epi formula was used for the baseline assessment of kidney function. This estimation may itself introduce bias in the prediction of later kidney function, relative to the true GFR. To the extent that the CKD-EPI formula may perform differently in the two cohorts, our results could have impacted. We also did not distinguish between polycystic kidney disease genotypes 1 and 2, and prediction performance could vary between these groups. Further the R^2^ is known to be sensitive to the range and variability of the data; thus apparent improvement in prediction performance based on a higher R^2^ in our validation cohort compared to the original development cohort should be interpreted with caution.

It should be noted that the Mayo Clinic prediction model development set used TKV assessed via the ellipsoid equation [12], while the present study used the gold standard TKV assessment by boundary method [24], which could introduce additional variability in prediction performance. However, a recent study assessed patient reclassified by the Mayo risk classification system resulting from these different TKV assessment method. The investigators found only a limited impact with a few patients reclassified mostly to lower risk categories [24].

A second aim of our study was to evaluate whether additional information regarding TKV change could improve the model prediction performance. Based on the results of the validation study and relatively large size of the development sample, we followed Steyerberg’s approach [25] and fixed all regression coefficients at their original values under the premise that re-estimation runs the risk of replacing reliable but modestly biased estimators with unbiased but unreliable ones [10]. Allowing only the coefficient for TKV to vary, we found that the R^2^ remained relatively unchanged when baseline TKV was replaced with measurements from the first two assessments. Further including all available TKV measurements, including a current TKV assessment, did not provide substantial improvement in the prediction performance that would justify the additional cost, time and effort of TKV measurement.

Strengths of our study include a patient population that was entirely independent of the Mayo Clinic data set, varying geographically, culturally and temporally from the original development cohort. In addition the Swiss ADPKD study has comprehensive follow-up with repeated measurements of kidney volume over time in a well-described cohort of untreated ADPKD patients at an early disease stage. The inclusion of recently enrolled patients as well as those with nearly 10 years of follow-up establishes generalizability across the patient population. Prediction models need to perform well in general ADPKD patient populations, as they are used for clinical decision-making.

## Conclusions

In conclusion, we found that the Mayo Clinic prediction model is an accurate tool to identify those at highest risk for rapid disease progression as defined by declining kidney function. The performance of the model was not substantially improved with by including additional TKV assessments, suggesting that follow-up TKV measurements may not be worth the cost and burden for the purposes of predicting progression. The Mayo prediction model may be a valuable tool for identifying patients for whom new treatments such as tolvaptan will provide benefits that outweigh the burden of side effects.

## Abbreviations

ADPKD: Autosomal dominant polycystic kidney disease
AIC: Akaike’s information criterion
CKD-EPI: Chronic Kidney Disease Epidemiology Collaboration
CRPS: Continuous ranked probability score
eGFR: Estimated glomerular filtration rate
HtTKV: Height adjusted total kidney volume
IQR: Interquartile range
MRI: Magnetic Resonance Imaging
TKV: Total kidney volume
